# A Mobile Phone App for the Prevention of Type 2 Diabetes in Malaysian Women With Gestational Diabetes Mellitus: Protocol for a Feasibility Randomized Controlled Trial

**DOI:** 10.2196/37288

**Published:** 2022-09-08

**Authors:** Madeleine Benton, Iklil Iman, Kimberley Goldsmith, Angus Forbes, Siew Mooi Ching, Iliatha Papachristou Nadal, Nicola Guess, Helen R Murphy, Barakatun-Nisak Mohd Yusof, Anisah Baharom, Nur Hafizah Mahamad Sobri, Nurul Iftida Basri, Mazatulfazura Sf Salim, Irmi Zarina Ismail, Faezah Hassan, Khalida Ismail, Boon How Chew

**Affiliations:** 1 Department of Psychological Medicine King's College London London United Kingdom; 2 Department of Family Medicine Universiti Putra Malaysia Selangor Malaysia; 3 Department of Biostatistics & Health Informatics King’s College London London United Kingdom; 4 Division of Care in Long-term Conditions King’s College London London United Kingdom; 5 Research Centre for Optimal Health University of Westminster London United Kingdom; 6 Department of Medicine University of East Anglia Norfolk United Kingdom; 7 Department of Nutrition and Dietetics Universiti Putra Malaysia Selangor Malaysia; 8 Department of Community Health Universiti Putra Malaysia Selangor Malaysia; 9 Department of Obstetrics and Gynaecology Universiti Putra Malaysia Selangor Malaysia; 10 Department of Rehabilitation Medicine Universiti Putra Malaysia Selangor Malaysia; 11 Clinical Research Unit Hospital Pengajar Universiti Putra Malaysia Serdang Malaysia

**Keywords:** gestational diabetes mellitus, diabetes prevention, randomized controlled trial, mobile app

## Abstract

**Background:**

Over 50% of women with a history of gestational diabetes mellitus (GDM) will develop type 2 diabetes (T2D) in later life. Asian women experience a disproportionate risk of both GDM and T2D compared to women from other ethnic backgrounds. Lifestyle interventions and behavior change can delay or even prevent the onset of T2D. We have developed a digitalized diabetes prevention intervention for the prevention of T2D in Malaysian women with GDM.

**Objective:**

The protocol describes a randomized controlled trial (RCT) to test the feasibility of undertaking a definitive trial of a diabetes prevention intervention, including a smartphone app and group support. Secondary aims are to summarize anthropometric, biomedical, psychological, and lifestyle outcomes overall and by allocation group, and to undertake a process evaluation.

**Methods:**

This is a two-arm parallel feasibility RCT. A total of 60 Malaysian women with GDM will be randomized in the antenatal period to receive the intervention or standard care until 12 months post partum. The intervention is a diabetes prevention intervention delivered via a smartphone app developed based on the Information-Motivation-Behavioral Skills model of behavior change and group support using motivational interviewing. The intervention provides women with tailored information and support to encourage weight loss through adapted dietary intake and physical activity. Women in the control arm will receive standard care. The Malaysian Ministry of Health’s Medical Research and Ethics Committee has approved the trial (NMRR-21-1667-60212).

**Results:**

Recruitment and enrollment began in February 2022. Future outcomes will be published in peer-reviewed health-related research journals and presented at national, regional, or state professional meetings and conferences. This publication is based on protocol version 2, January 19, 2022.

**Conclusions:**

To our knowledge, this will be the first study in Malaysia that aims to determine the feasibility of a digital intervention in T2D prevention among women with GDM. Findings from this feasibility study will inform the design of a full-scale RCT in the future.

**Trial Registration:**

ClinicalTrials.gov NCT05204706; https://clinicaltrials.gov/ct2/show/NCT05204706

**International Registered Report Identifier (IRRID):**

PRR1-10.2196/37288

## Introduction

### Background

Gestational diabetes mellitus (GDM) is one of the most common medical complications of pregnancy. The condition is defined as glucose intolerance resulting in hyperglycemia, which is first recognized in pregnancy and resolves after delivery [1]. Prevalence rates vary considerably from 1.8% to 31.5% depending on the diagnostic criteria used and the population studied [2]. Globally, prevalence is increasing, mirroring general upward trends in noncommunicable disease, maternal age, and obesity prevalence. In the short term, GDM is associated with obstetric complications including pre-eclampsia and cesarean section [3,4], and undesirable perinatal outcomes including large birth weight and shoulder dystocia [5]. In the long term, GDM is associated with increased maternal risk of GDM recurrence in subsequent pregnancies [6], cardiovascular disease [7], and type 2 diabetes (T2D) [8], while children of mothers with GDM are at increased risk of obesity, hypertension, and T2D later in life [9]. GDM is the single strongest population predictor of T2D. Approximately 50% of mothers with GDM will develop diabetes within 10 years [8,10,11].

People of Asian ethnicity are at higher risk of developing T2D than people of White ethnicity. More than 60% of people with T2D live in Asia [12,13] with prevalence rates estimated to increase by more than 150% between 2000 and 2035 [13]. Among Asian countries, Malaysia has one of the highest comparative prevalence rates of T2D at 20.8% [14] compared with other neighboring countries such as Singapore (12.8%) and Thailand (8%) [15]. Malaysia has a multiethnic society including three major ancestral groups (Malay, Chinese, and Indian), with Malaysian Indian people having the highest prevalence of T2D (28%), followed by Malay people (19%) and Chinese people (9%) [16]. Women in Malaysia also experience GDM at disproportionate rates in comparison to women from other ethnic groups. The incidence of GDM in Malaysia was 7.7% in 2016 and 9.3% in 2017 [17].

Evidence from landmark studies has shown that healthy lifestyle interventions and behavior change can delay or even prevent the onset of T2D. The Finnish Diabetes Prevention Study and the Diabetes Prevention Program both demonstrated a 58% reduction in the incidence of T2D in individuals with impaired glucose tolerance after 3 years of lifestyle interventions focused on diet and physical activity [18,19]. Similar findings have been observed in an Asian setting. The Da Qing Diabetes Prevention Study, after 6 years of lifestyle intervention, was associated with reduced incidence of T2D by 31%, 46%, and 42% in the groups of diet, exercise, and diet plus exercise, respectively [20], and benefits extended over 20 years after the intervention was discontinued [21].

However, evidence to support the efficacy of prevention interventions in women with a history of GDM is not clear. Subgroup analysis of the landmark Diabetes Prevention Program showed a 50% reduction in T2D incidence in women (n=350) with prior GDM in the previous 10 years [22]. Further, a meta-analysis of randomized controlled trials (RCTs) on the effectiveness of lifestyle interventions in the prevention of T2D in women with previous GDM demonstrated a 25% reduction in diabetes risk [23]. Small but statistically significant reductions in weight, BMI, and waist circumference were reported with longer periods of intervention [23]. Several barriers have been identified to lifestyle interventions in postpartum women with recent GDM, including tiredness, lack of time, competing work and family demands, childcare, and cultural expectations [24,25].

Mobile health (mHealth) is a rapidly growing field of public health, defined as the use of mobile phones and other wireless technology to support health objectives [26]. Due to the increasing ownership of smartphones, significant numbers of mHealth apps have been developed [27]. Pregnant and postpartum women are increasingly using such technologies as sources of health information and services for pregnancy self-care and infant care [28,29]. Smartphone apps can provide a novel way to deliver health interventions during this life stage, as they can address previously mentioned barriers experienced by women due to their flexibility and home-based approach.

### The Malaysian Gestational Diabetes and Prevention of Diabetes Study

The Malaysian Gestational Diabetes and Prevention of Diabetes Study (MY GODDESS) aims to reduce the risk of T2D in women with GDM in Malaysia. The project consists of two work streams. The first work stream involves a systematic review to synthesize process evaluations of RCTs on diabetes prevention interventions for women with current or a history of GDM [30], focus groups using qualitative methods to identify barriers and facilitators to uptake of DPI [31], focus groups to model the diabetes prevention intervention, and development of an interactive smartphone app. The second work stream involves a feasibility RCT of the developed diabetes prevention intervention for women with GDM in Malaysia. This paper presents the protocol for the MY GODDESS feasibility RCT.

The overall aim of the RCT is to test the feasibility of undertaking a definitive trial of a diabetes prevention intervention including a smartphone app and group support over 15 months in women with GDM from randomization in the antenatal period to 12 months post partum.

## Methods

This protocol was designed according to the SPIRIT (Standard Protocol Items: Recommendations for Interventional Trials) 2013. A SPIRIT study timeline (Table 1) and SPIRIT checklist (Multimedia Appendix 1) are provided.

**Table 1:** SPIRIT (Standard Protocol Items: Recommendations for Interventional Trials) timeline for the trial.

				Study period																		
				Enrollment		Randomization		Postrandomization											Ongoing			Close-out
				–t_1_		0		t_1_		t_2_		t_3_		t_4_		t_5_						
**Enrollment**																						
	Eligibility screen			✓^a^																		
	Informed consent			✓																		
	Allocation					✓																
**Intervention**																						
	Standard care + app					✓																
	Standard care					✓																
**Assessments**																						
	**Demographic**																					
		Date of birth			✓																	
		Ethnicity			✓																	
		Postcode of residence			✓																	
		Education level			✓																	
		Employment status			✓																	
		Household income			✓																	
		Gravidity			✓																	
		Parity			✓																	
		Previous pregnancy with GDM^b^			✓																	
	**Anthropometric**																					
		Body weight			✓		✓		✓		✓		✓		✓							
		Height			✓																	
		BMI			✓		✓		✓		✓		✓		✓							
		Body fat percentage			✓		✓		✓		✓		✓		✓							
	**Biomedical**																					
		Systolic and diastolic blood pressure			✓		✓				✓				✓							
		Total cholesterol			✓		✓				✓				✓							
		LDL^c^ cholesterol			✓		✓				✓				✓							
		HDL^d^ cholesterol			✓		✓				✓				✓							
		HbA_1c_^e^			✓		✓				✓				✓							
		Fasting plasma glucose			✓		✓				✓				✓							
		OGTT^f^ 2 hours postprandial									✓				✓							
		Insulin resistance (HOMA-IR^g^)			✓		✓				✓				✓							
	**Psychological**																					
		SEE^h^			✓						✓				✓							
		Maternal Antenatal Attachment Scale			✓						✓				✓							
		Maternal Postnatal Attachment Scale									✓				✓							
		PHQ-9^i^			✓						✓				✓							
	**Lifestyle**																					
		24-hour dietary recall			✓						✓				✓							
		SF-IPAQ^j^ score			✓						✓				✓							
		Step count			✓						✓				✓							
		Smoking status			✓																	
		Alcohol			✓																	
		Infant feeding practices									✓				✓							
**Clinical variables**																						
	**Maternal medical information**																					
		Hypercholesterolemia							✓													
		Hypertension							✓													
		Pre-eclampsia							✓													
	**Maternal pregnancy and birth data**																					
		Place of antenatal care							✓													
		Date of delivery							✓													
		Place of delivery							✓													
		Delivery method							✓													
		Pregnancy complications							✓													
		Birth complications							✓													
		Breastfeeding status							✓													
	**Neonatal data**																					
		Birth weight							✓													
		APGAR^k^ score							✓													
		Congenital anomaly							✓													
		Congenital pneumonia/heart defects							✓													
		Admission to neonatal intensive care, special care, or qualified on ward							✓													
		Length of stay							✓													
Feasibility outcomes																		✓				
Process measures										✓		✓						✓				

^a^Included at this time point.

^b^GDM: gestational diabetes mellitus.

^c^LDL: low-density lipoprotein.

^d^HDL: high-density lipoprotein.

^e^HbA_1c_: hemoglobin A_1c_.

^f^OGTT: oral glucose tolerance test.

^g^HOMA-IR: Homeostatic Model Assessment for Insulin Resistance.

^h^SEE: Self-Efficacy for Exercise.

^i^PHQ-9: Patient Health Questionnaire.

^j^SF-IPAQ: Short-form International Physical Activity Questionnaire.

^k^APGAR: appearance, pulse, grimace, activity, and respiration.

### Ethics Approval

Ethics approval was granted by Malaysian Ministry of Health’s Medical Research and Ethics Committee (NMRR-21-1667-60212). The study is registered on ClinicalTrials.gov (NCT05204706).

### Study Design

**Figure 1:**
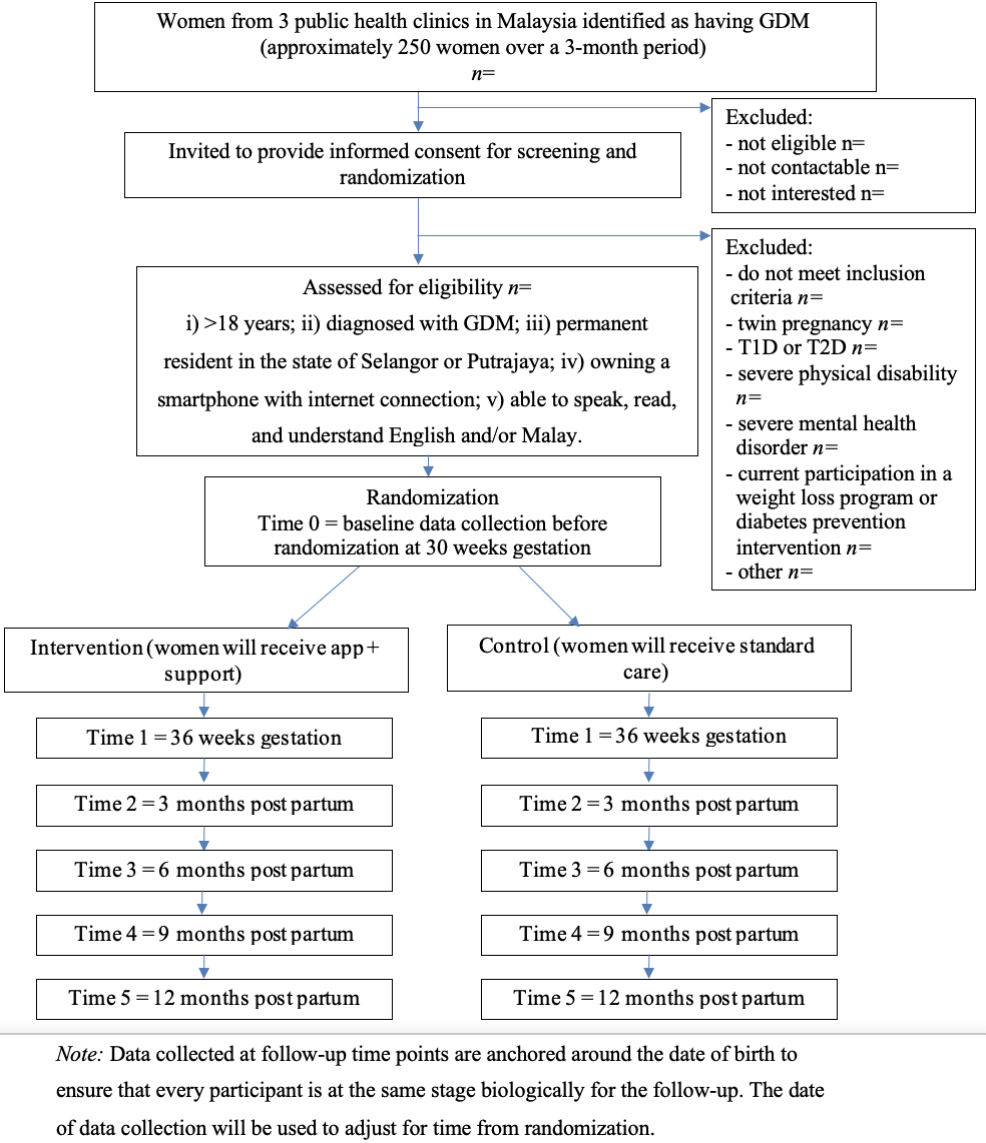
Study flow diagram. GDM: gestational diabetes mellitus; T1D: type 1 diabetes; T2D: type 2 diabetes.

This is a two-arm, parallel, open-label feasibility RCT with an allocation ratio of 1:1 for intervention and control arms. The two arms are the intervention arm comprising of a smartphone app and group support delivering a lifestyle intervention and a control arm consisting of standard care. The flow of participants through the study is presented in Figure 1.

### Study Setting

Participants will be recruited from three public health clinics within the states of Selangor and Putrajaya in Malaysia. Each clinic provides care for around 100,000 people. In these health clinics, the average total daily reported GDM cases range from 5 to 10 cases, with an annual prevalence of 700 to 1000 women.

### Eligibility Criteria

#### Inclusion Criteria

Women will be eligible for recruitment if they meet the following criteria: older than 18 years; diagnosed with GDM defined using fasting blood glucose >5.1 mmol/1 or 2-hour postprandial >7.8 mmol/1, which are the standard guidelines for diagnosis of GDM in Malaysia [32]; permanent resident in the state of Selangor or Putrajaya; registered in one of the study health clinics; owning a smartphone (iOS 11 or Android 7) with internet connection; and able to speak, read, and understand English or Malay.

#### Exclusion Criteria

Women will be excluded if they meet any of the following criteria: are having a twin pregnancy, have type 1 or 2 diabetes, have a severe physical disability that would prevent any increased uptake of physical exercise, have a severe mental health disorder (psychosis, bipolar, substance dependence, or active suicidal ideation), or are currently participating in a weight loss program or diabetes prevention intervention. For women who decline to participate, their age, parity, BMI, ethnic origin, education level, occupation, and household income will be recorded if permission is granted.

### Recruitment and Informed Consent

The study population will be all women with GDM in Selangor and Putrajaya, Malaysia. In Malaysia, pregnant women are screened for GDM in the first trimester (if they have risk factors) or in the second trimester between 24 and 28 weeks gestation using the 2-hour oral glucose tolerance test (OGTT) [32]. The results are recorded in an antenatal register. Potentially eligible women will first be identified from this register by clinic staff and introduced to a research team member. The research team member will provide women with a study brochure, a verbal description of the study, and an opportunity to ask questions about the study. Women will be given at least 1 week to consider their participation. If the participant agrees, informed consent and screening for eligibility to participate in the study will be obtained. Recruitment will be adapted to be conducted remotely depending on the nature of any COVID-19 restrictions in place at time of recruitment.

### Baseline Measures Collected Before Randomization

#### Sociodemographic

Date of birth, ethnicity, postcode of residence, education level, occupation, household income, gravida, parity, and number of previous GDM pregnancies.

#### Anthropometric

Body weight will be measured in light clothing, without shoes, to 0.01 kg, and height to 0.1 cm using a stadiometer. Height will be measured without shoes and socks using a stadiometer (SECA 213) to the nearest 0.1 cm. The BMI will be calculated as weight (kg) / height^2^ (m^2^). Body fat percentage will be assessed using bioimpedance analysis (Tanita RD-545 InnerScan Pro).

#### Biomedical

Systolic and diastolic blood pressure (mmHg) will be measured using a digital Omron HBP-1120 monitor using standardized procedures of the average of two readings taken 1 minute apart while seated. Blood venous samples will be collected via venipuncture. The lipid profile (total, low-density lipoprotein [LDL], and high-density lipoprotein [HDL] cholesterol) will be measured using an Advia 2400 (Siemens Diagnostics) analyzer, detection limit 0.1 mg/L and 0.01 mmol/L, respectively. Hemoglobin A_1c_ (HbA_1c_) level (mmol/mol) and fasting blood glucose (mmol/l) will be measured by affinity chromatography using the Primus Ultra 2 analyzer (Primus Corporation) and an Advia 2120 analyzer (Siemens Diagnostics), respectively. For the OGTT, participants will be asked to fast for 8 hours, after which fasting blood glucose and fasting insulin will be measured, then the participant will be invited to drink 75 g of glucose in 200 ml of water, and 2 hours later the blood glucose will be measured. The Homeostatic Model Assessment for Insulin Resistance (HOMA-IR) will be calculated by multiplying fasting plasma glucose (mmol/l) by fasting serum insulin (U/mL) divided by 22.5.

#### Psychological

Self-efficacy will be measured using the Self-Efficacy for Exercise (SEE) Scale, which comprises 18 items [33]. Response options are presented on a Likert scale from 0 to 100, ranging in 10-unit intervals from 0 (cannot do) through intermediate degrees of assurance, 50 (moderately certain can do), to complete assurance, 100 (highly certain can do). The scale is scored by summing the numerical ratings for each response and dividing by the number of responses. Higher scores indicate higher self-efficacy for exercise. The mother-infant relationship will be assessed in the antenatal period using the Maternal Antenatal Attachment Scale, which comprises 19 items with response options presented on a 5-point Likert scale [34]. Higher scores are indicative of stronger attachment, but a specific cutoff is not provided [34]. Depressive symptoms will be measured using the Patient Health Questionnaire (PHQ-9), comprising 10 items scored on a 4-point Likert scale with a score range from 0 to 27 and higher scores representing worse depressive symptoms and a score 10 representing caseness for diagnosis of depressive disorder [35].

#### Lifestyle

Dietary intake will be assessed using standardized multiple-pass 24-hour dietary recall for 3 days (2 weekdays and 1 weekend day) as it is considered an objective and reliable measure of change in intervention studies [36]. Clinical researchers will be trained to follow a standardized protocol, ask neutral probing questions to encourage recall of food items, and taught about different methods of food preparations and brands in different cultures. Physical activity will be measured using the Short-form International Physical Activity Questionnaire (SF-IPAQ), which comprises seven items to capture average daily time spent sitting, walking, and engaging in moderate and vigorous physical activity over the last 7 days [37]. Step count will be measured using inbuilt features of the patients’ own smartphone; the previous 7-day average will be used. Smoking status will be measured using a single item measure with response options including current smoker, previous smoker, and never smoked. Alcohol intake will be measured using the first question of the Alcohol Use Disorders Identification Test [38].

### Randomization

Women who meet study inclusion criteria and consent to study participation will be randomized using simple block randomization to two groups. The randomization system will be set up online using the system Sealed Envelope [39]. The randomization list will be generated by clinical research unit (CRU) officers prior to the first participant’s baseline visit at 30 weeks of pregnancy, using random permuted blocks of 2 and 4 to treatment A or B in a ratio of 1:1 to ensure that the groups are balanced periodically. Once the randomization list has been generated, access to the list will be password protected. Only CRU officers will have access to the list and will be responsible for randomizing participants. At 30 weeks of pregnancy during the baseline visit, once the consent form has been signed, eligibility has been confirmed, and baseline assessments have been completed, a research member in the clinic will call CRU officers to obtain group allocation. The CRU officers will access the randomization list the same day to obtain the allocation. Recruitment and randomization will be ongoing until each arm of the study comprises 30 participants.

### Trial Arms

#### Control Arm: Standard Care

Women allocated to the control arm will receive standard care and no intervention. This includes self-monitoring blood glucose and lifestyle advice (diet, physical activity, optimal body weight) by a multidisciplinary team.

#### Intervention Arm: Mobile App + Group Support

Women allocated to the intervention arm will receive access to a smartphone app called MyManis (the English meaning of MyManis is *my sweet baby)* and group support.

#### MyManis App

The development of the app was guided by the Information-Motivation-Behavioral Skills (IMB) model of behavior change [40]. The IMB model identifies three core determinants of the initiation and maintenance of health behaviors: accurate information that can be readily translated into health behavior performance (ie, knowledge), motivation both personal and social to act on such information, and behavioral skills to execute the health behavior effectively and confidently. The constructs of the model are supported in the literature to improve healthy lifestyle behaviors and have been tested and used successfully in diabetes management [41-43], obesity prevention [44], and improving dietary and physical activity behaviors [45,46].

Participants randomized to the intervention will receive a brochure outlining the app features and a step-by-step guide on how to download the app and set up an account. A link to download the app via SMS text message will also be sent to participants for convenience. Once the app has been downloaded, participants will be invited to set up an account by inputting their name, date of birth, current week of gestation, height, and weight. Once completed, this will give them full access to the app. The app content is presented under six main tabs:

1. Home page: featuring weekly motivational and educational blog posts
2. Information: comprising clinical information about GDM and T2D including definitions, causes, consequences, and management
3. Diet: providing women with information on healthy eating including understanding relative amounts of the three main macronutrients using the Healthy Plate Method and information on healthy food swaps. Women will be provided with a series of recipes that reflect Malay, Indian, and Chinese ethnicities. Recipes will be grouped into breakfast, lunch, and dinner, and will be tailored according to the participants’ nutritional requirements (based on BMI derived by the participant’s log in details).
4. Exercise: comprises a weekly program of exercise tailored to pregnancy and the postpartum stage. Exercise programs are presented using visual aids including images and short videos. The focus will be on increasing step count, as walking is the most culturally appropriate activity in Malaysia. Safety precautions, including pre-exercise screening, tips for before/after the exercise including warm-up and cooldown, and monitoring the exercise’s intensity using target heart rate calculation and Borg Scale are also incorporated in the app.
5. Well-being: providing information in relation to personal well-being in both the antenatal and postnatal period
6. GDM monitoring: allowing women to store personal information, blood glucose readings, and antenatal and postnatal appointment notes

The app is available to women in both Malay and English

#### Peer Support

Women will be invited to join group peer support. This will consist of 1-hour sessions with a group of 10 women meeting weekly via a virtual group chat function and communication of SMS text messages supporting each other during the week. Group support will be facilitated by a dietician trained in motivational interviewing (MI).

#### Technical Care

Women will be notified via SMS text message if an update of the app is required. During follow-up visits to their health clinic, women will be provided with in-person app support if required. Women who have not accessed the app for over a 2-week period will be flagged, and a notification will be sent via the app to motivate engagement.

### Data Collection

#### Feasibility Outcomes

The primary feasibility outcomes are to estimate the proportion of women who were randomized out of those identified from the clinical registers (study population); the proportion of women who take up the intervention out of those in the intervention arm, to be defined by measures of digital analytics (data will be collected on date of log in, time spent on each component of the app, and total time spent on the app); and the proportion of women who are withdrawn or lost to follow-up out of those randomized (ie, those who provide no primary or secondary outcome data).

The secondary feasibility outcomes are to estimate the proportion of women identified from clinical registers (study population) who give consent for screening for eligibility; the proportion of women who were randomized out of those eligible to participate; the proportion of those who completed the secondary outcomes at follow-up out of those randomized; and the number of women randomized per month overall and per month per study site.

#### Secondary Outcomes

##### Anthropometric

Anthropometric outcomes will be collected antenatally at 36 weeks gestation (time 1), and at 3 (time 2), 6 (time 3), 9 (time 4), and 12 (time 5) months post partum. They include weight (kg), height (cm; at time 1 only), BMI (kg/m2), and body fat percentage.

##### Biomedical

Biomedical outcomes will be collected antenatally at 36 weeks gestation (time 1) and at 6 (time 3) and 12 (time 5) months post partum. They include systolic and diastolic blood pressure (mm Hg), total cholesterol (mmol/L), LDL cholesterol (mmol/L), HDL cholesterol (mmol/L), HbA_1c_ (mmol/mol), fasting plasma glucose (mmol/L), OGTT (mmol/L; time 3 and 5 only), and insulin resistance (HOMA-IR).

##### Psychological

Psychological outcomes will be collected at 6 (time 3) and 12 (time 5) months post partum. They include self-efficacy (total SEE score), mother-infant relationship (total Maternal Postnatal Attachment Scale score), and depressive symptoms (total PHQ-9 score).

##### Lifestyle

Lifestyle outcomes will be collected at 6 (time 3) and 12 (time 5) months post partum. They include dietary intake (standardized multiple-pass 24-hour dietary recall), physical activity (total SF-IPAQ score), step count (mean steps, inbuilt smartphone function), and infant feeding practices (total score).

#### Process Evaluation Outcomes

A mixed methods six-component process evaluation framework will be used. Data collection methods for each component of the process evaluation are described below.

##### Recruitment

- Participant recruitment flow; number of eligible, consented, screened, enrolled, and randomized; reason for nonparticipation or exclusion
- Participant characteristics, sociodemographic data
- Recruitment strategy, description of recruitment pathways into MY GODDESS

##### Dose delivered

- Total number of MyManis app downloads
- Total number of group support sessions delivered

##### Dose received

- Digital analytics of MyManis app including date of log in, total number of log ins per month, length of log in, time spent on each component of the app
- Group support attendance logs

##### Program implementation/fidelity

- IT logs of the MyManis app will be collated on technical issues reported when using the intervention
- Assessment of competency of MI-trained dieticians in delivering group support

##### Provider experience

- Semistructured interviews with clinical research members who were responsible for participant recruitment and MI-trained dieticians who were delivering group support. Interviews will be conducted at the conclusion of the study and guided by an interview schedule.

##### Participant experience

- Semistructed interviews to understand participant experiences of the trial, satisfaction, and acceptability (women in the intervention arm only). Data will also be collected on the acceptability of assessing the mother-infant relationship via observation from participants perspectives at 3 months. Mother-infant relationships will be assessed via video recording obtained either at the clinic or via videoconference at the woman’s home. The recordings will aim to include both the mother’s and baby’s faces to enable coders to adequately visualize subtle movements, facial expressions, and vocalizations. Mothers will be advised to “talk and play with your baby as you normally would” prior to the recording. Recordings will be uploaded to a password‐protected computer and assessed and coded based on the Child Adult Relationship Experimental Index [47].
- Satisfaction survey: a purpose-designed survey regarding acceptability and perceived usefulness of the MyManis app

#### Clinical Variables

The following maternal and neonatal variables will be collected to describe the sample and the potential attrition and generalizability of a larger trial in this population. These variables will be collected at 3 months post partum (time 2) from postnatal records by a research team member. While we will not statistically assess them as such in this feasibility study, these variables may also be potential predictors or modifiers of postpartum outcomes at the later time points, which we may need to consider for a larger trial. These include maternal medical data (gestational hypertension, gestational hypercholesterolemia, pre-eclampsia); maternal pregnancy and birth data (place of antenatal care, date of admission, date of delivery, place of delivery, date of discharge, delivery method, pregnancy complications, birth complications, or breastfeeding status on discharge); and neonatal data (birth weight; appearance, pulse, grimace, activity, and respiration [APGAR] score 5 minutes; congenital anomaly; congenital pneumonia/heart defects; admission to neonatal intensive care; special care or qualified on ward; length of stay).

### Statistical Analysis

#### Sample Size

The aim is to recruit 30 women per arm, as this should be a large enough sample to estimate parameters such as the SD, which in turn can be used to estimate the sample size for an appropriately powered full-scale RCT [48]. This sample should also allow us to estimate the 95% CI for the primary feasibility outcome of the proportion randomized of those eligible, which we estimate will be approximately 10%, to a CI of within +/-8% [49]. This was calculated as:



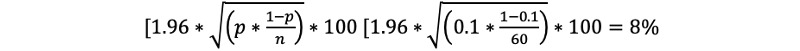



#### Plans for Statistical Analysis

Data will be double entered into an SPSS (IBM Corp) data set, and data analysis for the study will be performed using SPSS. Statistical analysis and reporting will be presented in line with the CONSORT (Consolidated Standards of Reporting Trials) guidelines [50]. Statistical analyses will primarily be descriptive, aiming to provide estimates related to feasibility parameters and to help inform power calculations for a future definitive trial.

For the primary outcomes of feasibility, we will calculate proportions or rates and appropriate CIs. We will consider whether a larger phase III effectiveness trial is feasible based on three potential factors: ≥25% of eligible women agree to be randomized, ≥50% of women in the intervention arm take up the intervention, or there is less than <20% attrition (women withdrawn from the study or lost to follow-up).

The secondary outcomes will be summarized using appropriate summary statistics overall and by randomization arm at each outcome time point. Normally distributed continuous variables will be summarized using the mean and SD, skewed continuous variables using the median and IQR, and categorical variables using frequencies and proportions. As the majority of the secondary outcomes are continuous and repeatedly measured, we will estimate the mean difference between the arms using linear mixed effects models with a random intercept at the participant level. The repeatedly measured outcomes will be the dependent variables, with the independent variables being trial arm, time, and a trial arm by time interaction term to extract differences at different time points. We will use analysis of covariance models where possible (ie, also include the baseline measure of the outcome as another independent variable). Appropriate 95% CIs will be calculated for the overall group and by trial arm, but as this is a feasibility study, we will not compare these statistically nor provide a *P* value for comparisons between arms. We will also not impute missing data, although fitting the mixed effects models using maximum likelihood will help account for missing data under a missing at random assumption. Some variables (eg, PHQ-9 and conversion to T2D [HbA_1c_ of 48 mmol/mol]) will also be summarized (but not analyzed) as categorical variables based on their clinical cutoffs. Smoking status and the obstetric outcomes that will only be measured once will also be summarized rather than modeled.

In terms of the process evaluation, all quantitative data including checklists, logs, and feedback surveys will be analyzed using SPSS. Data will be analyzed descriptively by calculating total numbers and percentages and mean and SD or, if the data are not normally distributed, the median and range. Qualitative data will be analyzed using thematic analysis to identify, analyze, and report patterns (themes) within the data [51].

Overall, the proportion of missing data for individual items and measures will be examined to determine the suitability of instruments and the level of burden for a future full-scale trial. As this is a feasibility study, no subgroup analyses are planned.

## Results

Recruitment and enrollment began in February 2022. Future outcomes will be published in peer-reviewed health-related research journals and presented at national, regional, or state professional meetings and conferences. This publication is based on protocol version 2, January 19, 2022.

## Discussion

### Overall Aim

The overall aim of this RCT is to test the feasibility of undertaking a definitive trial of a diabetes prevention intervention including a smartphone app and group support over 15 months in women with GDM from randomization in the antenatal period to 12 months post partum. Secondary aims are to summarize anthropometric, biomedical, psychological, and lifestyle outcomes overall and by allocation group, and to undertake a process evaluation. Prior to undertaking a definitive RCT of effectiveness, evidence is needed to determine if such a trial could be undertaken and confirm outcomes likely to be of the most importance in a future trial.

### Strengths and Limitations

To our knowledge, this is one of the few feasibility RCTs to test a smartphone app designed to prevent T2D by intervening prior to birth for women with GDM. It is also the first RCT we know of developed specifically for the prevention of T2D for Malaysian women with GDM. Further, the primary ethnic groups in this study (Malay, Chinese, and Indian) are also the major ethnic groups in Asia, and therefore, if this intervention proves to be effective in the larger RCT, this may also be applicable to the wider Asian population. The limitations are that this a feasibility study and therefore not powered to test effectiveness at this stage.

### Research and Clinical Implications

Findings from the feasibility RCT will assist in optimizing the diabetes prevention intervention smartphone app for a full-scale RCT. This will provide greater evidence and important knowledge in terms of diabetes prevention interventions using digital technology during this phase of a woman’s life, not only in Malaysia but also worldwide, which is crucial for slowing the rise of T2D.

## Appendix

Multimedia Appendix 1 SPIRIT (Standard Protocol Items: Recommendations for Interventional Trials) checklist.

Multimedia Appendix 2 Peer-review report 1 from the United Kingdom-Malaysia Health Research Partnership - UK Medical Research Council - Malaysia Partnerships and Alliances in Research (MyPAIR).

Multimedia Appendix 3 Peer-review report 2 from the United Kingdom-Malaysia Health Research Partnership - UK Medical Research Council - Malaysia Partnerships and Alliances in Research (MyPAIR).

Multimedia Appendix 4 Peer-review report 3 from the United Kingdom-Malaysia Health Research Partnership - UK Medical Research Council - Malaysia Partnerships and Alliances in Research (MyPAIR).

Multimedia Appendix 5 Peer-review report 4 from the United Kingdom-Malaysia Health Research Partnership - UK Medical Research Council - Malaysia Partnerships and Alliances in Research (MyPAIR).

## Abbreviations

APGAR: appearance, pulse, grimace, activity, and respiration
CONSORT: Consolidated Standards of Reporting Trials
CRU: clinical research unit
GDM: gestational diabetes mellitus
HbA_1c_: hemoglobin A_1c_
HDL: high-density lipoprotein
HOMA-IR: Homeostatic Model Assessment for Insulin Resistance
IMB: Information-Motivation-Behavioral Skills
LDL: low-density lipoprotein
mHealth: mobile health
MI: motivational interviewing
MY GODDESS: The Malaysian Gestational Diabetes and Prevention of Diabetes Study
NHS: National Health Service
NIHR: National Institute for Health and Care Research
OGTT: oral glucose tolerance test
PHQ-9: Patient Health Questionnaire
RCT: randomized controlled trial
SEE: Self-Efficacy for Exercise
SF-IPAQ: Short-form International Physical Activity Questionnaire
SPIRIT: Standard Protocol Items: Recommendations for Interventional Trials
T2D: type 2 diabetes
