# Supplementary material for: A Mobile Phone App for the Prevention of Type 2 Diabetes in Malaysian Women With Gestational Diabetes Mellitus: Protocol for a Feasibility Randomized Controlled Trial
Source: JMIR Res Protoc. 2022 Sep 8;11(9):e37288. doi: 10.2196/37288 (PMC9501684; doi:10.2196/37288)
Supplement: Multimedia Appendix 2 [file resprot_v11i9e37288_app2.pdf]

Medical Research Council
2nd Floor David Phillips Building, Polaris House, North Star Avenue, Swindon,
United Kingdom SN2 1ET
Telephone +44 (0) 1793 416200
Web http://www.mrc.ac.uk/

COMPLIANCE WITH THE DATA PROTECTION ACT 1998

In accordance with the Data Protection Act 1998, the personal data provided on this form will be processed by MRC, and may be held on computerised database and/or manual files. Further details may be found in the **guidance notes**

Research Grant
Peer Review

MRC Reference: MR/T018240/1

Document Status: With Council

UK-Malaysia Health Research Partnership

2019

Applicant Details

Applicant	Professor Khalida Ismail	Organisation	King's College London

Title of Research Project

The Malaysian GestatiOnal Diabetes and prevention of DiabtES Study (MY GODDESS)

Review Information

Response Due Date	16/08/2019	Reviewer Reference:	168733990

Research Quality

Research Quality: Please comment on the importance and competitiveness of the proposed research, including:

(1) strength of medical or scientific case (2) level of innovation, and whether this is likely to lead to significant new understanding (3) management strategy proposed, including equitable access to any shared resources (4) feasibility of experimental plans, statistics, methodology and design, including provision of sample size calculations, strategies to avoid bias, and preliminary data where appropriate (5) how well risks have been identified, and will be mitigated.

(1) Strength of scientific case

This proposal addresses an important topic for women in Malaysia who have a high prevalence of gestational diabetes mellitus (GDM) and subsequent high rates of type 2 diabetes. There have been several studies to date looking at lifestyle modifications to prevent type 2 diabetes, and none so far have replicated the very impressive reductions achieved in the US DPP.

The investigators have taken a very comprehensive approach to ensuring adequate understanding of the knowledge and capability of women and health providers, with attention to the modelling and development of the intervention. They have a strong track record and depth of experience in this area, which could make a value contribution to this field.

However what is somewhat unclear, and not well described is the intervention and the feasibility trial. The investigators state that they plan to implement a post partum diabetes prevention intervention (DPI), with recruitment at the end of the second trimester of pregnancy (24 weeks) and follow up until 12 months. They state there will be one face to face session, however it is unclear when and where this will occur. It is not clear when the motivational interviewing and other behaviour change techniques will be delivered and what relationship these will have with the App (will the App be the same for

everyone or will there be tailored messaging?). Will any additional intervention be delivered during the pregnancy to women in either arm of the trial? Where will the follow up visits occur and who will do the assessments? What will be the procedure if a woman is found to have type 2 diabetes during the follow up period of the trail or falls pregnant again? What will happen if the baby is stillborn or dies in the newborn period after recruitment?

(2) Innovation:
This work is likely to lead a moderate new understanding in the prevention of T2D after GDM, however given that this is feasibility work I think that is all that can be expected. The authors clearly state this is not a pilot RCT and they will not be comparing clinical endpoints.

(3) Management:
Both institutions have strong track records in managing research projects. I have no concerns about the management aspect of the proposal, although please see the section for resources .

(4) Feasibility:
I have some concerns about the ability to create and iteratively test an App in the short time period outlined in the study timeline (between months 9-10). If this was an adaptation of an existing App this may be possible, however from the description it appears this will be a bespoke App developed from the findings of study 1b and 1c.

I have some concerns about the feasibility particularly around the intervention development which is not described in detail. The authors state that they wish to measure several parameters such as physical activity and dietary intake, however it is unclear whether this will be using existing functionality on the women's smartphones, a proprietary 3rd party app or wearable device, and how this data will be securely stored and transmitted. In the description of the feasibility study it states that women will have accelerometer activity for 7 days pre randomisation. It is not clear whether this will be captured from women's own phones (in which case how accurate is this for measuring activity?) or by a separate device (which will need to be costed).

(5) Risks
The major risks I think will be around the clinical trial, with a risk that the start will be delayed because phase 1 is not completed or there are technical issues with the App development, or that recruitment will be slower than anticipated. There is also the possibility of delayed recruitment and loss to follow up. How the App relates to any existing national digital strategies in Malaysia and consideration of potential pathways to sustainability are also not addressed. This is a major limitation in scaling digital pilot studies

Research Environment and People

Please comment on the suitability of the investigator group and the environment where the proposed research will take place, including (1) track record(s) of the individuals in their field(s) and whether they are best-placed to deliver the proposed research (2) level of commitment of host research organisation to supporting the proposed research (3) whether appropriate facilities will be available to the researchers

The track records of the individuals seem appropriate for the proposed research. I note however that none of the investigators brings particular strength in digital health.

The host UK research organisation is very strong, with a solid track record in clinical trials and translation of research into practice. The Malaysian partner is also strong, however it is less clear from the application the experience in trial management, which I think from the application will be happening in the UK.
.
The Malaysian host organisation has indicated it's commitment to the program.

I am unsure whether the attachment on page 59 is meant to indicate commitment from KCL as it seems to be about another grant?

Impact

Please comment on the potential economic and societal impact of the proposed research, including (1) identification of realistic potential improvements to human or population health (2) contribution to relieving disease/disability burden and/or improving quality of life (3) identification of potential impacts of research and plans to deliver these (in the Pathways to Impact statement)

If this study is successful in demonstrating a culturally appropriate program to decrease type 2 diabetes after GDM in women, this would have future economic benefit for Malaysia and possibly other places. Directly from this feasibility work the impact will be less immediate.

The investigators state the approach will generate "a multitude of academic benefits across disciplines." The benefit directly to those involved in the study is less formally stated. The investigators mention economic benefits, however whilst there is a lot of focus on understanding process analysis in this application, there is not much emphasis on understanding and comparing the cost of interventions and assessing the cost of the intervention and the cost of sustaining it beyond development phase.

The two research groups are both well placed to communicate benefits and translate any findings in to policy, which is a plus.

Ethics

Please comment on any ethical and/or research governance issues, including (1) whether proposed research is ethically acceptable (2) any ethical issues that need separate consideration (3) appropriateness of ethical review and research governance arrangements (4) any potential adverse consequences for humans, animals or the environment and whether these risks have been addressed satisfactorily in the proposal

the proposed research is ethnically acceptable and there are plans for Malaysian and UK ethics review.

As stated in previous sections, provision will need to be made for women who experience stillbirth or neonatal death in the clinical trial.

When the protocol is developed it will be essential to have some consumer involvement in the writing and editing to make sure procedures are acceptable to women in Malaysia.

Data Management Plan

Please assess whether the data management plan indicates whether the applicants have (or are likely to have) a sound plan for managing the research data funded through the award, taking into account (1) the types, scale and complexity of data being (or to be) managed; (2) the likely long-term value for further research including by sharing data; and (3) the anticipated information security and ethics requirements.

(1) The plan is sound for the qualitative data, however it does not mention how data collected through the App will be collected and stored securely (for example will the App be password protected? how will the investigators ensure appropriate secondary use of this data?).

(2) The investigators have addressed potential long-term value of the data
(3) I am not convinced this has been adequately addressed: how will the App data be stored and backed up? where will it be stored and who will have access to it? How will data be extracted from clinical records?

Resources Requested

Please comment on (1) whether funds requested are essential and justified by the importance and scientific potential of the research (2) investigator time and proposed involvement related to management of the research (3) whether the proposal demonstrates value for money in terms of the resources requested (4) whether any animal use is fully justified in terms of need, species, number, conformance to guidelines

(1) Whilst the funds requested overall seem justified by what the investigators are trying to achieve, I am not sure whether the breakdown is reflective of the work to be done and I have concerns that they have over-costed some parts and not adequately costed others. For example whilst it is detailed that there will be costs for academic (post doc and post MSc) staff in Malaysia to coordinate the phases of the project, I can't see any costs for the third party App developer or cost of running the trial (CTU costs, research nurses, data entry, database set up etc) for the trial. I don't have access to a detailed breakdown of the costs from the Malaysia and UK groups, perhaps there has been a problem with the uploads? It is not clear from my reading of the application that these additional costs will be met by the partner organisations.

(2) The investigator time in relation to the management of the research: I found this quite confusing to appraise, as on page 9 it states that there will be 100% of full time spent on the project by the 5 Malaysian co-investigators, however there is no amount given and these names are marked with an asterisk, with no reference to the asterisk to explain what this means. In the justification of resources, the Malaysian team describe 2 post doctoral positions, 1 post-masters position, total of 5 trips for the co-Is to the UK, research materials (NB it is unclear from the rest of the application where the body fat composition will occur), and two tablet computers. They have budgeted £226417.40 for this, which seems excessive without a breakdown. If the Malaysian team are claiming 100% salaries for all co-investigators I don't think this can be justified by the proposal as it stands.

(3) If the investigators are able to deliver all the outcomes as planned, this would represent value for money, however as mentioned above I have concerns about the transparency and completeness of the costings.

Overall Assessment

Score 1-6

1 - Poor	2 - Good	✔ 3 - High	4 - Very High	5 - Excellent	6 - Exceptional
