# Supplementary material for: A Mobile Phone App for the Prevention of Type 2 Diabetes in Malaysian Women With Gestational Diabetes Mellitus: Protocol for a Feasibility Randomized Controlled Trial
Source: JMIR Res Protoc. 2022 Sep 8;11(9):e37288. doi: 10.2196/37288 (PMC9501684; doi:10.2196/37288)
Supplement: Multimedia Appendix 3 [file resprot_v11i9e37288_app3.pdf]

Medical Research Council
2nd Floor David Phillips Building, Polaris House, North Star Avenue, Swindon,
United Kingdom SN2 1ET
Telephone +44 (0) 1793 416200
Web http://www.mrc.ac.uk/

COMPLIANCE WITH THE DATA PROTECTION ACT 1998

In accordance with the Data Protection Act 1998, the personal data provided on this form will be processed by MRC, and may be held on computerised database and/or manual files. Further details may be found in the **guidance notes**

Research Grant Peer Review

MRC Reference: MR/T018240/1

Document Status: With Council

UK-Malaysia Health Research Partnership

2019

Applicant Details

Applicant	Professor Khalida Ismail	Organisation	King's College London

Title of Research Project

The Malaysian GestatiOnal Diabetes and prevention of DiabtES Study (MY GODDESS)

Review Information

Response Due Date	21/08/2019	Reviewer Reference:	162617988

Research Quality

Research Quality: Please comment on the importance and competitiveness of the proposed research, including:

(1) strength of medical or scientific case (2) level of innovation, and whether this is likely to lead to significant new understanding (3) management strategy proposed, including equitable access to any shared resources (4) feasibility of experimental plans, statistics, methodology and design, including provision of sample size calculations, strategies to avoid bias, and preliminary data where appropriate (5) how well risks have been identified, and will be mitigated.

1. The is a medically and scientifically sound proposal. The MRC framework for complex interventions will be used, which is appropriate for the proposal. This proposal has all the good characteristics of implementation research as the investigators in depht are examing what might and might not work before planning and performing a pilot RCT.
2. There is a high Level of innovation, as motivational inverviewing, phone Messages, Electronic teaching sessions and an app all we be considered.
3. The management strategi proposed is well thought through. Resources are allocated as needed.
4. The proposal has planned the required staff and funding. There is a lot to be done in just two years. The pilot RCT will "only" include 50 patients and with the large numbers of women With GDM described in Malaysia this is probably done very quickly. However, even a pilot RCT requires a lot of work and as does the Development of an app. However, some preliminary work has already been done and this is an experienced team. Still it will remain a challenge for the team to do all they have proposed within the 24 months.
5. There is no Clear identification of risk, nor how they will be mitageted. However, the team seems to have a very good insight in the settings, the requirements and how to deal with those.

Research Environment and People

Please comment on the suitability of the investigator group and the environment where the proposed research will take place, including (1) track record(s) of the individuals in their field(s) and whether they are best-placed to deliver the proposed research (2) level of commitment of host research organisation to supporting the proposed research (3) whether appropriate facilities will be available to the researchers

1. The team is multidiciplinary With a good mixture of highly experienced researchers and clinicians and individuals who are gaining experience. The track record of the the PI shows that she is experienced in leading and completing Projects. The members of the team have Strategic placements and several of them have worked together on Research Projects before. All in all a highly qualified team, well placed to perform the proposed Research.
2. The proposal includes a supporting letter from the University Putra Malaysia signed by the deputy director showing knowledge of the project and the required support. the supporting letter from King's College London confirms the financial support to the project. The researchers from that university have been there a while suggesting good working conditions and sufficient support.
3. Appropriate facilities appear to be available.

Impact

Please comment on the potential economic and societal impact of the proposed research, including (1) identification of realistic potential improvements to human or population health (2) contribution to relieving disease/disability burden and/or improving quality of life (3) identification of potential impacts of research and plans to deliver these (in the Pathways to Impact statement)

Gestational diabetes and subsequent Type 2 diabetes are obviously major Public health issue in Malaysia affecting the health of many women at a relatively Young age. The intensive and multi-faceted intervention is very likely to make an impact. In particular when health care professionals currently are not advising any lifestyle changes to women With gestational diabetes. So the potential benefits for many individuals of this Project is enormous. The question is to what degree the intervention can be scaled up when it it so intensive as proposed. Some elements of the intervention such as an app require the same maintenance irrespective of the number of people using the app. Motivational interviewing, sending mobile telephone messages however are labour intensive and may not be able to be scaled up. The potential impacts of the Research and plans of how to deliver these have been identified and described in detail.

Ethics

Please comment on any ethical and/or research governance issues, including (1) whether proposed research is ethically acceptable (2) any ethical issues that need separate consideration (3) appropriateness of ethical review and research governance arrangements (4) any potential adverse consequences for humans, animals or the environment and whether these risks have been addressed satisfactorily in the proposal

The proposed Research is ethically acceptable. There are no issues that need seperate consideration. Ethical approval will be sought both in the UK and Malaysia. Participants will be under close medical supervision while participating in the RCT.

Data Management Plan

Please assess whether the data management plan indicates whether the applicants have (or are likely to have) a sound plan for managing the research data funded through the award, taking into account (1) the types, scale and complexity of

data being (or to be) managed; (2) the likely long-term value for further research including by sharing data; and (3) the anticipated information security and ethics requirements.

The data managment plan indicates which data will be collected and how and how the different types of data will be analysed With the help of the appropriate data analyses programs. Storage and sharing of data is described. Possible risks have been identified and rightly so been deemed minimal due to the use of study ID and operating procedures. Sharing this data has been considered after a 5 year period during which the study team has exclusive use of the data.<br>Participants will be asked for informed consent to share data either identifiable of not.

Resources Requested

Please comment on (1) whether funds requested are essential and justified by the importance and scientific potential of the research (2) investigator time and proposed involvement related to management of the research (3) whether the proposal demonstrates value for money in terms of the resources requested (4) whether any animal use is fully justified in terms of need, species, number, conformance to guidelines

Both the UK and Malaysia are Foreign countries for me, limiting my chances of assessing the funding sought for. However, the funding includes the expected personnel, meetings, travels, consumeables. The average time proposed used on the Project by the investigator and other collaborators seems reasonable. I cannot judge if the proposal demonstrates value for Money. No animal use is proposed.

Overall Assessment

Score 1-6

1 - Poor	2 - Good	3 - High	4 - Very High	✔ 5 - Excellent	6 - Exceptional
