# Supplementary material for: A Mobile Phone App for the Prevention of Type 2 Diabetes in Malaysian Women With Gestational Diabetes Mellitus: Protocol for a Feasibility Randomized Controlled Trial
Source: JMIR Res Protoc. 2022 Sep 8;11(9):e37288. doi: 10.2196/37288 (PMC9501684; doi:10.2196/37288)
Supplement: Multimedia Appendix 4 [file resprot_v11i9e37288_app4.pdf]

Medical Research Council
2nd Floor David Phillips Building, Polaris House, North Star Avenue, Swindon,
United Kingdom SN2 1ET
Telephone +44 (0) 1793 416200
Web http://www.mrc.ac.uk/

COMPLIANCE WITH THE DATA PROTECTION ACT 1998

In accordance with the Data Protection Act 1998, the personal data provided on this form will be processed by MRC, and may be held on computerised database and/or manual files. Further details may be found in the **guidance notes**

Research Grant Peer Review

MRC Reference: MR/T018240/1

Document Status: With Council

UK-Malaysia Health Research Partnership

2019

Applicant Details

Applicant	Professor Khalida Ismail	Organisation	King's College London

Title of Research Project

The Malaysian GestatiOnal Diabetes and prevention of DiabtES Study (MY GODDESS)

Review Information

Response Due Date	30/07/2019	Reviewer Reference:	143339203

Research Quality

Research Quality: Please comment on the importance and competitiveness of the proposed research, including:

(1) strength of medical or scientific case (2) level of innovation, and whether this is likely to lead to significant new understanding (3) management strategy proposed, including equitable access to any shared resources (4) feasibility of experimental plans, statistics, methodology and design, including provision of sample size calculations, strategies to avoid bias, and preliminary data where appropriate (5) how well risks have been identified, and will be mitigated.

The subject matter is indeed relevant and contemporary. The concept and idea is not novel but has not been explored adequately in Malaysia. The plan of study is well laid out. There are other researchers particularly in public health in Malaysia who have done extensive work and even copyrighted exercise protocols to prevent gestational diabetes mellitus. However, my main concern is a lack of IT expertise in the team of researchers, as development of an IT Application is an important output from this research, which is to be tested in the end.

Research Environment and People

Please comment on the suitability of the investigator group and the environment where the proposed research will take place, including (1) track record(s) of the individuals in their field(s) and whether they are best-placed to deliver the proposed research (2) level of commitment of host research organisation to supporting the proposed research (3) whether appropriate facilities will be available to the researchers

The study is proposed to be conducted in a research university, a fertile environment for productive research. It would be good to also harness the involvement of other universities, especially one with an established teaching hospital.

Impact

Please comment on the potential economic and societal impact of the proposed research, including (1) identification of realistic potential improvements to human or population health (2) contribution to relieving disease/disability burden and/or improving quality of life (3) identification of potential impacts of research and plans to deliver these (in the Pathways to Impact statement)

Obesity and diabetes are indeed important health hazards to be addressed in Malaysia, particularly among pregnant mothers. It is certainly pertinent to do a root cause analysis as to why it seems difficult to eradicate. Prevention of diabetes in pregnancy in Malaysia will certainly be a boon to the health standards of the country, with significant relief in disease burden. However, the risks and obstacles involved in effecting this is not clearly addressed in the proposal.

Ethics

Please comment on any ethical and/or research governance issues, including (1) whether proposed research is ethically acceptable (2) any ethical issues that need separate consideration (3) appropriateness of ethical review and research governance arrangements (4) any potential adverse consequences for humans, animals or the environment and whether these risks have been addressed satisfactorily in the proposal

Ethical approval is required and should not be a problem as the ethical risk is low.

Data Management Plan

Please assess whether the data management plan indicates whether the applicants have (or are likely to have) a sound plan for managing the research data funded through the award, taking into account (1) the types, scale and complexity of data being (or to be) managed; (2) the likely long-term value for further research including by sharing data; and (3) the anticipated information security and ethics requirements.

The team has a biostatistician on board and should not encounter any problems handling the data, which is not anticipated to involve a huge sample.

Resources Requested

Please comment on (1) whether funds requested are essential and justified by the importance and scientific potential of the research (2) investigator time and proposed involvement related to management of the research (3) whether the proposal demonstrates value for money in terms of the resources requested (4) whether any animal use is fully justified in terms of need, species, number, conformance to guidelines

The funding required may not necessarily be as large as requested. In this era of online communication, there does not seem to be a need for two week-long travels per researcher from the UK to Malaysia.

Overall Assessment

Score 1-6

1 - Poor	2 - Good	3 - High	✔ 4 - Very High	5 - Excellent	6 - Exceptional
