# Supplementary material for: A Mobile Phone App for the Prevention of Type 2 Diabetes in Malaysian Women With Gestational Diabetes Mellitus: Protocol for a Feasibility Randomized Controlled Trial
Source: JMIR Res Protoc. 2022 Sep 8;11(9):e37288. doi: 10.2196/37288 (PMC9501684; doi:10.2196/37288)
Supplement: Multimedia Appendix 5 [file resprot_v11i9e37288_app5.pdf]

**Medical Research Council**
2nd Floor David Phillips Building, Polaris House, North Star Avenue, Swindon,
United Kingdom SN2 1ET
**Telephone +44 (0) 1793 416200**
**Web http://www.mrc.ac.uk/**

**COMPLIANCE WITH THE DATA PROTECTION ACT 1998**
In accordance with the Data Protection Act 1998, the personal data provided on this form will be processed by MRC, and may be held on computerised database and/or manual files. Further details may be found in the **guidance notes**

# Research Grant Peer Review

MRC Reference: MR/T018240/1

Document Status: With Council

UK-Malaysia Health Research Partnership

2019

**Applicant Details**

| | | | |
|---|---|---|---|
| Applicant | Professor Khalida Ismail | Organisation | King's College London |

**Title of Research Project**

| |
|---|
| The Malaysian GestatiOnal Diabetes and prevention of DiabtES Study (MY GODDESS) |

**Review Information**

| | | | |
|---|---|---|---|
| Response Due Date | 12/08/2019 | Reviewer Reference: | 089498832 |

**Research Quality**

Research Quality: Please comment on the importance and competitiveness of the proposed research, including:

*(1) strength of medical or scientific case (2) level of innovation, and whether this is likely to lead to significant new understanding (3) management strategy proposed, including equitable access to any shared resources (4) feasibility of experimental plans, statistics, methodology and design, including provision of sample size calculations, strategies to avoid bias, and preliminary data where appropriate (5) how well risks have been identified, and will be mitigated.*

Gestational diabetes is the single biggest predictor of type 2 diabetes in women. It is commoner in some ethnic groups including Malaysians. There is now over 15 years real-world experience of diabetes prevention through lifestyle modification. Research to date has found it much harder to reduce the risk of diabetes among women who have had GDM than those who have not. While innovative, this proposal is muddled because the authors have not familiarised themselves sufficiently with the current state of knowledge. It has been known since the US DPP subgroup analysis (Ratner) that women who have GDM are far less amenable to lifestyle modification than those who have not. Two groups with extensive experience of diabetes prevention have shown how difficult it is to engage post GDM women and outcomes achieved in middle age appear not achievable in this group. (Ferrara, O'Reilly.) At this stage there is inadequate evidence to support the development of delivery by an app.

The researchers do not appear to have appreciated the difference between advice on diet for women who have GDM and women who have delivered and require a diabetes prevention diet. During pregnancy the aim is a diet which keeps blood glucose low. The only evidence for a diabetes prevention diet comes from the five goals of the Finnish Diabetes Prevention Study. There is evidence that this not only prevents diabetes but reduces cardiovascular risk. E.g. Retinopathy.

It follows that intervening during both pregnancy and after delivery requires two different programs. A recent systematic review in BMJ makes it clear that we do not need any further research on GDM prevention and reduction.

The proposal seeks to use the MRC complex intervention methodology. Although developments are not always linear, they roughly occurr in five stages. First the theory, second the modelling, third an exploratory trial, fourth a randomised controlled trial followed by long-term implementation (scaling-up). In this proposal, there is contingency between each of these stages. Applicants should complete stages one and two if not three before requesting funding for other stages.

They do not appear to have anyone experienced in real-world implementation (scaling up to system level) and the commentary about how it will be done in the fifth stage is quite naïve. There seems to be no appreciation that conversion to T2D from GDM requires a systems approach: screening all women, registering them, re-calling them for screening tests and diabetes prevention planning, and all the levers incentives required to make this happen.

There seems to be something of a mismatch between the team in London and the Malaysian team. The Malaysian team is largely from family medicine but no academic general practitioner is in the link role in London. Tertiary specialists from a high income country  don't match well with primary care physicians from a middle income country.

There is no convincing risk management plan.

**Research Environment and People**

*Please comment on the suitability of the investigator group and the environment where the proposed research will take place, including (1) track record(s) of the individuals in their field(s) and whether they are best-placed to deliver the proposed research (2) level of commitment of host research organisation to supporting the proposed research (3) whether appropriate facilities will be available to the researchers*

In general, this team brings together a large range of disciplines both at Kings and in Malaysia. Many of the investigators have highly distinguished track records in diabetes, gestational diabetes and diabetes education for self-management.

Specifically, there is no expertise in diabetes prevention not even post GDM. How the team interacts between London and Malaysia is unclear. There is no academic GP link in London and no health services implementation researcher.

The commitment at Kings and in Malaysia seems sound. I would expect a good level of support from the organisations.

**Impact**

*Please comment on the potential economic and societal impact of the proposed research, including (1) identification of realistic potential improvements to human or population health (2) contribution to relieving disease/disability burden and/or improving quality of life (3) identification of potential impacts of research and plans to deliver these (in the Pathways to Impact statement)*

I am sceptical about the likelihood of success because a) the team has no one experienced in diabetes prevention b) preventing conversion from GDM to T2D is the most difficult challenge in diabetes prevention.

I would have expected the proposal to refer to either NICE guidelines or EU IMAGE guidelines for diabetes prevention, or have included one of the British teams with expertise. E.g. Khunti in Leicester or Greaves in Birmingham.

Dunkley has shown the relationship between application of these guidelines and diabetes prevention outcomes in 'real world' settings. The nub of the guidelines would be text saying something like: facilitating participants to achieve the five

goals of the Finnish Diabetes Prevention Study by applying the Health Action Process Theory, problem-solving and goal setting, and self-determination theory.

**Ethics**

*Please comment on any ethical and/or research governance issues, including (1) whether proposed research is ethically acceptable (2) any ethical issues that need separate consideration (3) appropriateness of ethical review and research governance arrangements (4) any potential adverse consequences for humans, animals or the environment and whether these risks have been addressed satisfactorily in the proposal*

Ethics is satisfactory

**Data Management Plan**

*Please assess whether the data management plan indicates whether the applicants have (or are likely to have) a sound plan for managing the research data funded through the award, taking into account (1) the types, scale and complexity of data being (or to be) managed; (2) the likely long-term value for further research including by sharing data; and (3) the anticipated information security and ethics requirements.*

Satisfactory

**Resources Requested**

*Please comment on (1) whether funds requested are essential and justified by the importance and scientific potential of the research (2) investigator time and proposed involvement related to management of the research (3) whether the proposal demonstrates value for money in terms of the resources requested (4) whether any animal use is fully justified in terms of need, species, number, conformance to guidelines*

I am unable to assess UK/Malaysian costs.

**Overall Assessment**

*Score 1-6*

| | | | | | |
|---|---|---|---|---|---|
| 1 - Poor | ✔ 2 - Good | 3 - High | 4 - Very High | 5 - Excellent | 6 - Exceptional |
